# Inspiring rural youth to consider healthcare careers through an interprofessional healthcare traveling roadshow

**DOI:** 10.3389/fpubh.2024.1401805

**Published:** 2024-07-19

**Authors:** Kristjan Mytting, Martin Muermann, Sean B. Maurice

**Affiliations:** ^1^Division of Medical Sciences, University of Northern British Columbia, Prince George, BC, Canada; ^2^Department of Cellular and Physiological Sciences, Faculty of Medicine, University of British Columbia, Vancouver, BC, Canada

**Keywords:** rural, health workforce, interprofessional, healthcare, university-high school outreach, community engagement, youth

## Abstract

There are well-documented shortages of healthcare providers in rural and remote communities worldwide, and these shortages correlate with inequitable health outcomes for rural peoples. Despite a wide array of efforts to remedy the issue, these shortages persist to this day. The Healthcare Traveling Roadshow (HCTRS) is a grassroots initiative that began in 2010 to help address the shortage of healthcare providers in rural communities throughout British Columbia. Since its inception, the HCTRS has been predicated on three evidence-based guiding principles which have been shown to markedly increase the rate at which healthcare students choose to practice rurally. These principles are: (1) to showcase healthcare careers as viable and realistic options for rural youth (high school students) using interactive stations and near-peer teaching; (2) to expose healthcare students to rural communities and showcase them as a potential opportunity for their future practice; and (3) to provide a unique interprofessional experience to healthcare students from diverse healthcare careers and backgrounds. Through the synergy of these three principles the HCTRS aims to increase the longitudinal recruitment and retention of healthcare workers in underserved rural communities. This paper will share our experience from 15  years of running this initiative, for those hoping to implement similar programs in other areas of the world.

## Introduction

Providing access to adequate and equitable healthcare services for rural and Indigenous communities is a long-standing and well documented issue (1–4). Healthcare provider shortages in rural regions correlate with increased morbidity and mortality suffered by rural and Indigenous peoples, the world over (3–5). While approximately 17.8% of Canadians live in rural and/or remote locations (6), only 8.2% of practicing physicians are located within these areas (7). This disparity becomes even more staggering when looking specifically at specialist physicians, only 2.2% of whom practice in a rural setting (7). This shortage is not isolated to physicians either; many other healthcare professions are significantly underrepresented such as occupational therapists and physical therapists, with 5.3 and 7.2% practicing rurally, respectively, (8, 9). This maldistribution of healthcare providers and disparity in the provision of healthcare services, specifically in highly remote locations, generally leads to worse health outcomes due to delayed care, the need for lengthy transport, a higher financial/logistical burden, and longer recovery time (10).

In many ways rural youth are the best candidates for a career as a rural healthcare provider, as they are familiar with the culture and context of rural living (11, 12). However, rural youth face greater barriers than their urban counterparts in studying to be a healthcare provider, including needing to travel long distances, and typically needing to adapt to the urban environment, without nearby family support (13–15). Additionally rural youth, have greater hesitancy about their abilities to succeed in healthcare training (15–17).

Rural youth are less likely to have connections to people who work in healthcare and thus have less ‘social capital’ (17). Yet when youth have greater health-related self-efficacy, this contributes to social capital, and helps individuals and their peers consider the possibility of something that previously felt out of reach (17). Healthcare student facilitators, particularly those with a rural background and/or experience, are uniquely capable of engaging high school students through their relatability and role modeling (18). Thus, providing support and encouragement for rural youth to consider healthcare careers is an important aspect of building and sustaining the rural health workforce.

The Canadian public believe in the importance of universally accessible healthcare, but recognize that our health system is struggling significantly (19). People recognize the importance of growing the primary care workforce, and ensuring that this workforce is representative of the people it serves; which includes increasing training opportunities for northern, rural, remote, and Indigenous peoples (19). The Canadian Collaborative Taskforce for Advancing Rural Family Medicine has acknowledged that initiatives need to be developed, whose central goal is the longitudinal recruitment and retention of healthcare workers to rural and remote locations (1). The Canadian accrediting body for medical schools has recently added an accreditation element on social accountability that requires a medical school to address the priority health concerns of the populations it has a responsibility to serve (20). Given the established maldistribution of healthcare providers and the impact it has on the health of rural and Indigenous peoples, medical schools thus have an obligation to deliver outreach to rural, remote and Indigenous, youth (high school students).

This paper will describe and elucidate the logistics of delivering one such initiative, the Healthcare Traveling Roadshow (21, 22).

## The healthcare traveling roadshow

The Healthcare Traveling Roadshow (HCTRS) was conceived in 2009 as a grassroots initiative with the intent of lessening healthcare provider disparities in rural communities throughout the province of British Columbia, Canada (21, 22). In order to achieve this goal, the HCTRS recruits healthcare students from various training programs across a variety of healthcare career training programs, to take part in a 1 week activity visiting several communities within a specific, underserved region of British Columbia, Canada.^1^ These students are tasked with being part of a unique presentation and learning experience wherein they share their story with youth at local schools and engage them in interactive education stations focusing on diverse healthcare careers, having low stakes conversation and stimulating interest. This program serves to correct misconceptions about the possibility of pursuing a career in healthcare as well as providing youth with insight into various options potentially available to them and the scope of practice of specific healthcare professions. In addition to this, the healthcare student facilitators are exposed to rural communities, both in formal educational tours and recreational activities which showcase the natural beauty these areas contain and the potential benefits of practicing in such a setting. By touring facilities and speaking to local healthcare professionals, students are exposed to the reality of the available services and what’s possible in a resource limited setting. This increases the general understanding of rural healthcare service delivery, along with its challenges and opportunities, as well as showcases the opportunities and lifestyle inherent to practicing in these communities.

Over the past 15 years, 23 Roadshows have taken place which have visited more than 13,500 youth in 79 different community visits. These communities are vastly separated in their geographic location, but all share some measure of rural health service disparity. By promoting healthcare careers to rural youth, the HCTRS is designed to alleviate these service shortages by increasing the number of local youth choosing to pursue careers in healthcare, promoting rural practice to students in training, and providing an interprofessional experience for the healthcare students. Through the synergy of its three principles, the HCTRS provides a multifaceted approach to the longitudinal recruitment and retention of rural healthcare providers.

## Three guiding principles

From its inception, the HCTRS has been predicated on three guiding principles. While each could be perceived as an isolated, achievable goal; it is the unison of these principles which best serves to promote a change in the rural workforce (21). These principles are to:

1. Showcase healthcare careers as options for rural students.
2. Showcase the rural community as a career option for healthcare students.
3. Provide an interprofessional experience for the healthcare students.

Through ensuring that these core principles are met, the HCTRS is able to achieve consistent positive impact despite changing student facilitators and communities each year. In addition, as the HCTRS has gained a positive reputation, more communities have expressed a desire to have this program come to their region. This has prompted the creation of two additional annual roadshows. Currently, two annual trips are based out of Prince George, B.C. and explore communities within the north. The third is based out of Kelowna, B.C. and provides exposure to rural communities within the Southern Interior region. Given this distributed model, having these well-articulated principles assures communities that they will receive the same program regardless of their location. All three principles should be clearly understood as each provides an evidence-based approach that individually has been shown to improve rates of recruitment and retention of rural healthcare professionals (23).

### Showcase healthcare careers as options for rural students

Evidence shows that healthcare students with rural backgrounds are, on average, approximately twice as likely to return to and practice in a rural setting following the completion of their training (24). Evidence also suggests that obtaining secondary, post-secondary or post-graduate education in a rural setting likewise predisposes healthcare providers to practice rurally (1, 23, 24). Thus, showcasing healthcare careers to rural youth should eventually increase the number or rural healthcare practitioners. However, rural youth often experience numerous barriers in pursuing a path in healthcare, compared to students in more urban environments (13–15). Such obstacles include a lack of exposure to the healthcare system, consequent lack of familiarity with the multitude of diverse career options available, and far fewer role models within the healthcare field amongst many others. Through their interactions with rural youth, the healthcare student facilitators provide rural youth with exposure to numerous professions within the healthcare field and highlight these professions as realistic options for their future careers. The HCTRS uses a “near-peer” model, that may be more effective than having individuals who are more established in their careers’ talk to youth (25–28). In addition to benefitting the learners, near-peer teaching approaches have benefits for the personal and professional development of the peer teachers (28). This is the beginning of what has been described as the rural healthcare “pipeline” or “pathways;” concepts which seek to address the many barriers faced by underrepresented populations into healthcare training (1, 29).

### Showcase the rural community as a career option for healthcare students

Rural exposure is an important factor in a future healthcare practitioner’s decision to work and live in such a setting. It has been shown that having positive experiences in rural communities significantly increases the rates at which students choose to practice rurally (30). Unfortunately, many healthcare training programs within British Columbia have historically only been made available in metropolitan areas and offer little, if any, rural exposure during the course of training. Compounding this problem, the urbanization of healthcare training often leaves students without the experiences, skills, and training necessary to practice in rural setting should they choose to explore such an option later-on (31). As such it is vital that all healthcare students gain a measure of rural competence during their education even if they choose to practice in an urban environment. The rural exposure students are given during the HCTRS serves the dual purpose of improving students’ ability to practice in a rural setting, and increasing their likelihood of choosing to practice rurally. Should they ultimately choose to practice in a larger metropolitan area, their experiences will have better informed them concerning the challenges of rural healthcare delivery and their competence will better serve them in their future practice when engaging patients from a rural background.

### Provide an interprofessional experience for the healthcare students

Interprofessional experiences during the pre-clinical education of healthcare students helps them synergize as part of a larger healthcare team (31). While some programs require interprofessional activities as part of their training, others do not. Interprofessional collaboration is important for successful clinical practice, and even more important in rural contexts (32). Rural practice requires interprofessional collaboration, as healthcare providers must work closely with one another to mitigate gaps in service availability and provide the best possible care with limited infrastructure, staff, and resources.

Healthcare student facilitators are given an in-depth interprofessional experience during the HCTRS with fellow students from a diverse selection of healthcare training programs, and may also have the chance to meet rural healthcare providers within the visited communities. The healthcare students learn with, from, and about each other and must collaborate to carry-out the presentations and modify their approaches as needed (if seeing a different age group, if the initial presentation wasn’t working well). Throughout this process, the students gain a greater understanding of their professional role, gain experience describing their career and scope of practice to the public, and learn about the scope of practice of other healthcare careers, at a stage of training when it is perhaps easier to ask questions that might sound naïve (“what do you do?”). Ensuring healthcare students gain greater experience in this area while in training through projects like the HCTRS may lead to graduates being better prepared for work in a rural setting. Furthermore, some may find they naturally gravitate to a work environment containing a more prevalent component of interprofessional collaboration causing them to more seriously consider work in a rural setting.

## Impact

To date, our program evaluation has indicated that the program is largely successful in reaching its goals. Youth, healthcare students, and community members alike, agreed that the program was largely successful and succeeded in encouraging rural youth to consider a career in healthcare, while showcasing the rural community as an appealing option for healthcare students to consider (Table 1) (22). Respondents emphasized that the sincerity of the presenters and interactive design of the initiative made it impactful; community engagement is important; the interprofessional design is important for the youth to see as well as for the healthcare students to experience; and healthcare students can learn much through participating in such an initiative (Table 2) (22).

**Table 1:** Quantitative evaluation of the healthcare traveling roadshow.

		Youth (95% CI)	Community members (95% CI)	Healthcare students (95% CI)	Total (95% CI)
Q1.	How successful do you think this project was in general? 1 = Not very, 5 = Very	4.68	4.68	4.75	4.71
		(4.44–4.92)	(4.55–4.81)	(4.64–4.86)	(4.63–4.79)
Q2.	Do you think this project will encourage healthcare students to consider settling in a rural community after graduation? 1 = Not likely, 5 = Very likely	4.14	3.66	4.58	4.12
		(3.84–4.44)	(3.46–3.86)	(4.43–4.73)	(3.98–4.26)
Q3.	Do you think this project has encouraged local youth to consider careers in healthcare? 1 = Not much, 5 = Very much	4.55	4.31	4.54	4.45
		(4.30–4.80)	(4.15–4.47)	(4.39–4.69)	(4.35–4.55)
Q4.	Please rate your overall experience of the project. (Healthcare students only) 1 = Poor, 5 = Excellent			4.82	4.82
				(4.71–4.93)	(4.71–4.93)

Average of responses to program evaluation questions, with 95% confidence intervals (CI), presented by population: youth (*n* = 22), community members (*n* = 62), healthcare students (*n* = 57), and total (*n* = 141 or *n* = 57 total for Question 4). Reproduced with permission under Creative Commons CC-BY license (22).

**Table 2:** Highlights of program evaluation.

Sincerity and Interactivity Sparking Enthusiasm
“Everything was pretty great. I liked that everything was hands on, and that we got to learn a bit about everyone.” – Youth
“I think the range of backgrounds/life experiences/career choices each student had was great for kids to see.” – Community member
“Inspiring and educating the high school students… Seeing their “aha” moments and enthusiasm.” – Healthcare student
“All the hands-on work that we were able to see. I liked how all the medical students personalized their work.” – Youth
“The interactive tables were great for kids to actually see what might be involved, as many of these kids have probably never been exposed to most of the professions incorporated in the roadshow.” – Community member
“I liked the connection obtained by talking to the people who had come for my sake.” – Youth
Learning Through Rural Exposure and Community Engagement
“Having healthcare students from all over BC … allows us to showcase our community and what we have to offer professionals coming to the [region].” – Community member
“It is inevitable that students will all encounter patients at some point in their career who have come from a smaller community and have faced some of the health care challenges that comes along with that. Having at least some idea of what those challenges are will allow students to be better equipped to care for these patients.” – Healthcare Student
“I can genuinely say that because of this opportunity, I would consider working in a more rural area as a healthcare professional.” – Healthcare Student
“The Roadshow gave me incredibly valuable insight into what this sort of medical path would be like and I feel like firsthand exposure is really the only way to obtain this sort of insight.” – Healthcare Student
Healthcare Student Personal Growth
“It’s great to learn what brought people to their career of choice and it’s inspiring to know there are lots of options to be shared with not only youth but anyone looking for a rewarding career.” – Healthcare Student
“Learned about how to effectively describe my own profession to others, learned about the role/scope of other fields.” – Healthcare Student
“Talking to the students from other training programs helps me to communicate with other health professionals better in my future work.” – Healthcare Student
“That personal sense of brining this important cause to a region that requires this work so acutely and desperately was satisfying.” – Healthcare Student
Interprofessional Collaboration and Development
“…I realized while traveling to these small towns that teamwork is key. It can be daunting and overwhelming to manage the health of an entire community, which is why support networks and interprofessional cooperation among different health professionals is vital for long-term sustainability.” – Healthcare Student
“I learned a little more about what is involved in the different professions as well, which makes it easier for me to understand how I can incorporate partnerships later on when I am in practice.” – Healthcare Student
“I was impressed with the depth of knowledge across all different disciplines and how much we can learn from each other and how our work with patients connects.” – Healthcare Student
“[I] learned that everyone brought a lot to the table and was able to gain more insight into how other peoples’ programs worked and it broadened my own understanding of a healthcare team” – Healthcare Student
“… the most enjoyable part of the roadshow has been learning from the other students that have been alongside me on the tour. I have found it incredibly informative to learn about the professions that influence healthcare in the clinical setting.” – Healthcare Student

Representative quotes organized by theme. Reproduced with permission under Creative Commons CC-BY license (22).

We are also aware of a number of stories of students who on admission to medical school, told us that the HCTRS was what planted the seed that led to their decision to pursue a career in medicine. We also know of at least one student who chose a career in medical laboratory technology science after seeing the HCTRS as a high school student and has since returned to their hometown and has remained there in practice. Many times on the HCTRS, healthcare students have had conversations about job opportunities with local medical staff. Much anecdotal evidence also indicates that the HCTRS has a dramatic impact on the number of conversations about healthcare careers at schools, for months after our visits.

## Funding

Funding for the HCTRS has grown as the initiative has grown, from a humble grassroots initiative, to a much larger initiative that still retains its grassroots approach. We have been very fortunate to benefit from stable funding from the provincial Rural Education Action Plan (33) and a local community trust, the Northern Medical Programs Trust (34) along with other partners as outlined on the HCTRS webpage (22). It is worth noting, that this initiative was not developed as a research project and has not been supported by research funds. There is a paucity of literature on theory and best practices for this type of initiative and more research should be done, but this initiative was developed with guidance from the literature that clearly indicates this type of work needs to be done. We feel it’s been as successful as it has, because we have focused on making the project successful, not on running it as a research project and needing to continuously seek out new sources of funding. This initiative fits well within the scholarship of application as described by Boyer (35, 36).

As the initiative has grown, the budget has grown accordingly. Early trips relied on healthcare students from the small university and college in the northern region of the province where the HCTRS was born. However, we consistently had trouble getting 12 students to volunteer for a trip, and we were not able to showcase a great diversity of healthcare careers. In 2015, we requested additional funds to support students traveling from the large metropolitan regions in the south (i.e., Vancouver, Victoria) and this has allowed us to get a consistent pool of applicants representing more diverse healthcare careers and more diverse life experiences.

## Community selection

When running the HCTRS, it is important to have community buy-in. When we first started the HCTRS, we had a model that relied on a community champion in addition to an academic champion (i.e., staff or faculty lead). The community champion would help to coordinate with the school, the hospital, and local leaders, to provide a well-rounded experience for the healthcare student team. If a community champion volunteered because they knew about the HCTRS, the process was likely to be relatively easy. If a community champion did not volunteer or was not aware of the initiative, it tended to be harder to explain what it was about and required more effort from the academic champion. More recently, medical students have been organizing the trips, and we are largely a known entity in our region, but still, when speaking with someone new, who does not know what the initiative is, it can take substantial time and effort to explain what it is about, and to move them from seeing this as a chore to seeing it as a gift.

## Logistics

Each trip is different. In our rural region, trips can be over 2000 kms in a week, and we now charter a bus as a safer way (than personal vehicles) to carry the team and the equipment. The target audience is grade 10 students (mid-upper secondary education, ISCED level 3) (37), since they are expected to be in the early stages of considering future education and career opportunities, when “career-life interests and possibilities start to become meaningful considerations” for many (38). Depending on the size of the community and school population, we may see only grade 10 students or we may see the whole high school: i.e., if there are 240 youth from grade 8–12, we will likely plan 3 presentations and see the whole school; whereas, in a larger small town, if there 400 grade 10 students, we will only see grade 10 (in 5 different presentations).

We will occasionally visit an elementary school, though this is outside our target grade: if they are particularly remote, if we are invited, or if it otherwise seems like the right thing to do. It can be a fun way to get ‘warmed up’ for the high schools, as elementary school students tend to be easy to engage, and high school students aren’t necessarily. Also, if there is a very small rural high school near to a larger rural center, it can be fun for our students to see the small school, but there’s a tradeoff, as it takes time to get there, and get set up, do the presentation, and take down the equipment. If the school is very small (<30 students) we generally invite them to bus in to the larger school when we already have our equipment set up. This means less change of scenery for the healthcare students, but usually the youth from the small school enjoy the chance for a field trip.

A key component of the HCTRS is the availability of healthcare training equipment for the youth to interact with. This requires trusting relationships within the host institution(s) and any partnered institutions, to ensure this expensive equipment can be borrowed and used for the trip. In northern British Columbia, we borrow equipment from several UNBC programs: the medical program, nursing, nurse practitioner, and health sciences. We also borrow equipment from the local college (The College of New Caledonia) to model medical laboratory technology sciences and medical radiography technology. We also occasionally borrow equipment from the hospital as needed and available, such as an ultrasound or a patient monitor. If students are coming from a program we have not had before and/or do not have equipment for, we work with them to identify needs and will reach out to our local connections, and/or they may bring some supplies with them (their own, and/or borrowed from the school they are training at).

*A typical trip:*

- 1,000–2,500 kms traveled over a week.
- 3–6 different communities.
- 12 healthcare students representing 8–10 different healthcare careers.
- 2–4 university staff (admissions coordinator, communications officer, Indigenous coordinator, faculty lead).
- 10 presentations:
  o 60–80 youth per presentation (it’s important to not try to see too many students at once, or the small table presentations are too crowded and not everyone can see and engage/interact).
  o Each presentation includes ~12 min of large group introduction where each healthcare student has 1 min and 1 PowerPoint slide to answer the question “who am I, and why did I choose to become a [insert healthcare career]?” (youth sitting in bleachers or on the floor).
  o Following the large group introduction, healthcare students are divided into groups of ~6–8 and assigned tables to start at (Supplementary Appendix 1).
  o Small group presentations are meant to be interactive and give the youth something to do (intubate a dummy, look through a microscope at something interesting, try out a wheelchair, learn how to birth a baby, dress a wound, etc. …). They also provide an informal opportunity for youth to ask healthcare students questions, which vary from questions about biology related to the presentation, or questions about admission to the training program and costs of study. Youth often learn things through this exchange: about the availability of student loans, or that the placenta must be birthed after the baby, for example.
  o Preferred total presentation time 1.25–1.5 h (depending on schedule and flexibility at each school).
  o One of the staff is responsible for assigning youth to starting tables, and then must calculate the time available divided by the number of tables at the session (typically 6–8 min per station), and then keep time and tell the youth to rotate (sometimes this means cutting off conversation, but if we do not do this, youth will not get a chance to see every table and when that happened in the past, they were not happy about it).
- The 10 presentations can be 2 per day (morning is probably best), or can be scheduled more variably. Healthcare students consistently tell us that interacting with the youth is the most meaningful part of the trip; but that it’s also pretty exhausting.
- The remainder of the week schedule is filled with a combination of:
  o Hospital/healthcare facility tours: typically 2–3 facilities along the way. A larger rural hospital and a smaller one, to give the students an idea of the scope of positions available, and the range of facilities. Seeing multiple of the same size hospital has little additional value. Involving healthcare providers if possible is helpful, but often they are busy and the Health Services Administrator (HSA) often does the tour. The HSA tends to be quite knowledgeable about what positions are available, number of beds, services provided, etc.
  o Recreational opportunities. These depend widely on what the communities would like to showcase. We do not have a budget for recreation, so we try to ask community leaders if there are particular things that they’d like to showcase for this visiting group of healthcare students (a rather uncommon opportunity), to give them a taste of rural living. Sometimes the community will recommend a hike and send someone to guide us. Sometimes they will provide something more extravagant. Over the years we have done/seen: hiking, beach fires, canoe trips, horseback riding, a trip to the farmers market, whitewater rafting, helicopter tours, jet boat tours, museum tours, hot springs, a wildlife reserve, a very large ranch, an abandoned mine, swam in a backyard pool, been serenaded by the youth band, and more.
- The trips typically start Sunday with a commute to the initial community, Monday – Friday are presenting and visiting in different communities, then Saturday return home (7 days). For those who are traveling from outside the region, they will typically fly on Saturday and spend the night in the host community prior to the start of the trip, then at the end of the trip, they will generally spend another night before flying home on Sunday (9 days).

## Healthcare student recruitment

By bringing together an interprofessional team of healthcare student facilitators, the HCTRS serves to provide a unique interprofessional experience to these healthcare students unlike anything available today in traditional training programs (at least in our region), while simultaneously providing rural communities with new insight into the variety of healthcare professions and career opportunities that exist within the modern healthcare landscape. Wide recruitment efforts targeting students from diverse fields of healthcare is key to ensuring both of these goals are met. Recruitment across diverse programs and institutions is complicated however, in that students in different training programs often have schedules that may conflict or overlap regarding previous education commitments and clinical placements. The HCTRS now recruits students from any healthcare program and any institution in the province of BC.

Recruitment begins between 4 and 6 months prior to the trip(s). We have an application form that is built in Qualtrics (QualtricsXM^2^) (Supplementary Appendix 2), and we rank and adjudicate applicants, to ensure students understand what the HCTRS is about (it’s not just an opportunity to see rural communities). Applicants are scored using a rubric (Supplementary Appendix 3). Several medical students are involved in adjudicating the applications, and their scores are compiled, prior to an adjudication meeting. At the adjudication meeting, the faculty, staff and medical student team, review the scores, and then select students based on their scores, their careers, their diversity, and the trips they have chosen to attend. We aim for students with good scores, who represent diverse careers, and if possible diverse institutions and diverse lived experiences. We have funding for half of the student participants to travel from outside the region, so we aim for 6 students from our local institutions, and 6 from anywhere in the province. We aim for 8–10 different careers represented between the 12 total students (including 2 medical students who are involved in planning), and to have diversity in the lived experience of the group members. We tend to have >80% of applicants who are female, so we try to ensure there are some male students as well. If applicants are gender diverse, or disclose any differences in socioeconomic status, or ability (e.g., ADHD, anxiety, physical disability), they get additional points for diversity. It is particularly important in our region to role model Indigenous students in healthcare career training, as we have a high Indigenous population and not nearly the same percentage of healthcare providers who are Indigenous as the percentage of the population, so if applicants disclose Indigeneity, that is looked on positively as well.

Once a preliminary list of participants in created, the students are sent a welcome message and asked to confirm attendance within a week. If they can no longer commit, we reach out to the next best fit student. If they need longer to commit (to confirm with a preceptor that they can take time out of a placement, for example) we do our best to accommodate. Once we have a finalized list of students, they are sent more information on the trip (Supplementary Appendix 4), including the participant information form (Supplementary Appendix 5).

## Planning throughout the year

We begin planning our destinations in the fall, for the next spring (Supplementary Appendix 6). Experience has taught us that high schools need lots of warning, as they have many activities in their students’ schedules, and they aren’t able to accommodate something like this at the last minute (or even with 6 weeks’ notice). In the early years we would brainstorm possible communities, based in part on expressed community interest. Now that we have been to all the small communities in our region, we are planning repeat trips based on how long ago we were at each community. We finalize the schools and communities by December, so that in January we can begin recruiting students from other healthcare career training programs, knowing where the trips will be going.

After the adjudication process is complete and the students are selected for each trip, the remainder of the schedule needs to be planned/confirmed. The final schedule should include all activities each day including meals, the presentations at each school, time for setting up and taking down the presentations, recreational activities, local healthcare facility tours, checking in/checking out of hotels, and all transportation/travel time between each individual entry. Accommodations should be booked and confirmed several months in advance as smaller communities may have limited hotel accommodations available for a large group. Additionally, rural areas often have resource dependent economies that go through ‘boom and bust’ cycles, which greatly affect the availability of accommodations, as well as the costs. It has been found that having two students of the same gender share a room is preferable as this often allows friendships to form and saves costs. However, adjustments may need to be made depending on the participants.

Another consideration is to communicate with local restaurants well in advance to ensure they can accommodate larger group sizes. Just as with the hotel accommodations, food venues in smaller communities may not be able to easily accommodate large groups without warning. It is also important to ensure that restaurants can accommodate the dietary needs of the group. During the Roadshow, if the timing is tight, it can help to pre-order food so that dining can happen in the allocated window of time.

We do not have a budget for recreation. We ask our contacts and community leaders if there are particular things that they’d like to showcase for this visiting group of healthcare students, to give them a taste of rural living. It can help to remind them that this is an interprofessional group of healthcare students with some expressed interest in rural practice, and it’s an uncommon opportunity for them to be able to showcase their community to such a diverse group of healthcare trainees.

Plentiful demonstration equipment is essential to providing interactive small group presentations. Securing equipment for each individual student based on their unique clinical training and interests will maximize high school student engagement. As above, much equipment needs to be borrowed, and relies on having trusting relationships with different departments, institutions, and the local hospital. Examples of the type of equipment that can be brought, is in Supplementary Appendix 7.

A few months prior to the trips, a poster can be sent out to schools, to advertise that the Roadshow is coming to their school (Supplementary Appendix 8). An important element of planting the seeds of possibility, is to prepare students for the visit, and to hope that their parents/friends know about it and ask about it afterwards. The more conversation that happens about the possibility of a career in healthcare, the better.

On the trip itself, the days tend to be tiring, but also invigorating. Traveling together gives lots of opportunity for informal interprofessional sharing and for friendships to form. Though the students who volunteer to come on the trip have self-selected for the adventure, they may still be a bit shy at the start as they get to know one another. They will likely have a wide variety of experiences with youth (from none to plenty). If feasible, giving a chance to talk about their presentations the night before the first presentation (Sunday night dinner), can help to ease any concerns. After the first day of presentations, there will likely be some conversation about how it went, and more experienced students/staff can offer guidance, but in general, guidance should be minimal, so students can figure out how they wish to deliver their presentation (for some this is a new and somewhat scary experience).

Lastly, it’s important to include program evaluation, to ensure ongoing quality improvement of the initiative. The data from the spring trips can be reviewed at the initial planning meetings the next fall, to consider any changes in policy for the next year’s trips.

## Conclusion

The Healthcare Traveling Roadshow is a Northern British Columbia initiative that aims to reduce disparities in the provision of healthcare within rural communities. Healthcare student facilitators engage rural high school students and showcase their careers as realistic possibilities, in a hands-on, near-peer format that encourages questions. In addition, healthcare students are given a unique exposure to rural communities showcasing the realities and potential benefits to practicing in such a setting following the completion of their training. This is all achieved with an interprofessional focus, which is particularly important for the rural health workforce (31).

It is important to reiterate that this is not a research project by nature, though it is well supported by research as outlined above. This initiative fits well within the ‘scholarship of application’ and faculty interested in delivering such an initiative, may wish to be thoughtful about how they articulate this work on their CV’s. It is essential we do this work, with recommendations for national and international bodies concerned with the health of rural peoples, yet the academy is slow to accept the recommendations of Boyer and others (35, 36).

The HCTRS hopes to inspire similar initiatives in other areas that could benefit from a comparable program. This paper serves to share our framework for the implementation of a such an initiative, and we encourage anyone interested in developing their own version of this program to contact us if they require more information.

## Data availability statement

The original contributions presented in the study are included in the article/Supplementary material, further inquiries can be directed to the corresponding author.

## Ethics statement

Written informed consent was obtained from the individual(s), and minor(s)’ legal guardian/next of kin, for the publication of any potentially identifiable images or data included in this article.

## Author contributions

KM: Conceptualization, Methodology, Visualization, Writing – original draft. MM: Methodology, Writing – review & editing. SM: Conceptualization, Funding acquisition, Methodology, Project administration, Supervision, Writing – review & editing.

## Footnotes

^1^Jobs in healthcare outside of medicine are often collectively referred to as Allied Health Professions; however, some jobs in healthcare require college training for a certificate or diploma, not a university degree, and people in these positions may feel excluded by the term “profession,” thus we have chosen to refer to jobs in healthcare as “Healthcare Careers,” and the people doing these jobs as “Healthcare Providers,” to be inclusive.

^2^https://www.qualtrics.com
