# Supplementary material for: Inspiring rural youth to consider healthcare careers through an interprofessional healthcare traveling roadshow
Source: Front Public Health. 2024 Jul 19;12:1401805. doi: 10.3389/fpubh.2024.1401805 (PMC11295277; doi:10.3389/fpubh.2024.1401805)
Supplement: Supplementary file 1 [file Data_Sheet_1.docx]

**Inspiring rural youth to consider healthcare careers through an interprofessional healthcare traveling roadshow**

**Kristjan Mytting, Martin Muermann, Sean B. Maurice**

Supplementary Appendices

**Appendix 1: Sample station layout**

**
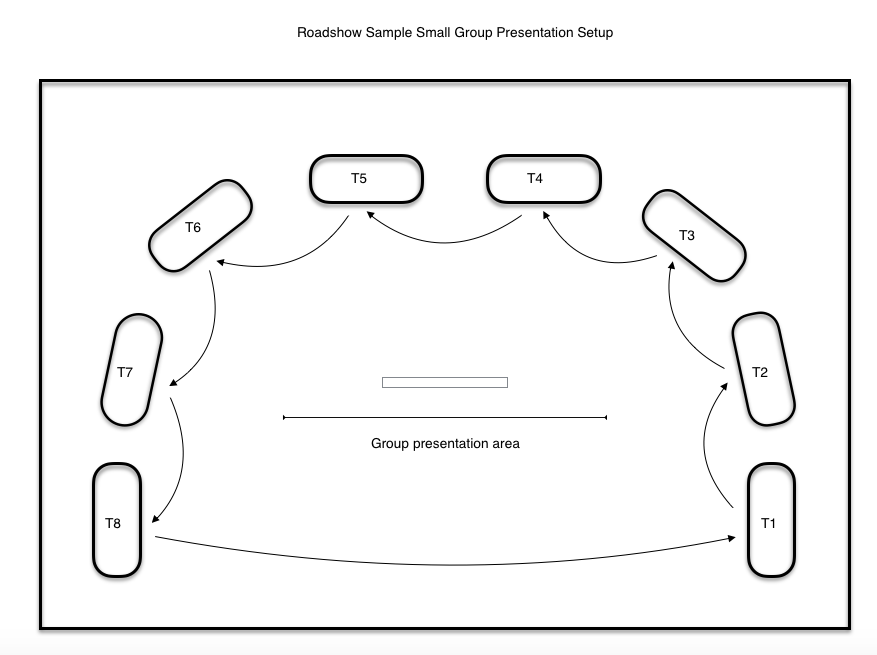
**

**Appendix 2: Sample Recruitment survey**

**2024 Healthcare Travelling Roadshow Application**

The Healthcare Travelling Roadshow was conceived as a grassroots initiative to address rural healthcare workforce shortages. It involves a multidisciplinary group of healthcare students from post-secondary institutions around B.C. travelling to rural communities to showcase career opportunities to high school students.

Since its inception in 2010, the roadshow has connected with more than 13,500 high school students during 79 community visits throughout BC.

If you are a healthcare student or recent graduate interested in participating in one of the 2024 roadshows, please submit your application by February 27, 2024.

For more info, visit <https://www2.unbc.ca/healthcare-travelling-roadshow>.

Please note: UBC Medicine students can only participate in the roadshow as part of the FLEX (Flexible and Enhanced Learning) course. UBC Physical Therapy, UBC Occupational Therapy, and UBC Dentistry students must obtain approval from their clinical placement coordinator prior to applying.

**First Name:**

**Last Name:**

**Gender:**

**Email Address:**

**Phone Number:**

**Program and Year of Study (e.g. Nursing, Year 2):**

**Post-Secondary Institution:**

**I am interested in participating in the following 2024 Roadshows (check all that apply):**

☐  Kootenay Roadshow (Castlegar, South Slocan, Kaslo): April 28 – May 4, 2024

☐  Peace Roadshow (Valemount, McBride, Mackenzie, Chetwynd, Fort Nelson): April 28 – May 4, 2024

☐  Nechako Roadshow (Vanderhoof, Fort St. James, Dease Lake, Stewart, Hazelton): May 12 – 18, 2024

If you ranked more than one Roadshow, please list your order of preferences (e.g. Peace #1, Kootenay #2, Nechako #3):

**Why are you interested in participating in the Healthcare Travelling Roadshow?** (max. 150 words)

**What motivated you to pursue your own healthcare career?** (max. 150 words)

**How will you contribute to the diversity of the roadshow team?** Please consider the question broadly and share anything you feel makes you a unique applicant. (max. 100 words)

**If you were selected to participate in the roadshow, what hands-on activity would you propose for your station to engage rural high school students?** (max. 100 words)

**Have you lived in or near any of the communities that the Healthcare Travelling Roadshow will be visiting this year? If so, where?**

**Have you been on a previous Healthcare Travelling Roadshow?**

☐  Yes

☐  No

**It is expected that applicants are able to commit to participating for the full duration of the roadshow trip. If you have any reservations about the timing of the trip(s), please explain:**

**Where did you hear about applying for the Healthcare Travelling Roadshow?**

Thank you for applying to the 2024 Healthcare Travelling Roadshow. We will begin reviewing applications shortly after the application deadline. Please note all applicants will be notified by mid-March. If you have an questions, please contact xxx@unbc.ca

**Appendix 3: Student Scoring Rubric**

**Healthcare Travelling Roadshow Scoring Rubric**

**1 2 3 4 5 6 7 8 9 10**

**| |**

**poor              excellent**

***Please input total scores (/25) for each applicant into Group Scoring Spreadsheet into your assigned column.***

| **Criteria** | **Score** |
| --- | --- |
| **Rationale for participation in HCTRS**  (for example: interest in and understanding of Roadshow mission and values including inspiring students, rural community engagement, and interprofessional teamwork) | **1   2   3   4   5   6   7   8   9   10** |
| **Rationale for choosing healthcare career**  (for example: Wanting to help people, improve patient quality of life or make a difference in their community or the global landscape) | **1   2   3   4   5   6   7   8   9   10** |
| **Diversity**  (for example: ethnicity, Indigenous, gender, socio-economic disadvantage) | **1   2   3   4   5** |
| **Total score** | **/25** |

**Appendix 4: Student Information Package**

**Healthcare Travelling Roadshow**

**Student Information Package**

**Travel to/from Prince George**

*If you are driving from outside Prince George:* You will be reimbursed for mileage at $0.62/km.

*If you are flying:* We will book your flight for you. Please let us know your preferred flights in the attached form. Remember to keep your receipts for transportation to and from the airport – we will reimburse you for this.

*If you are taking alternate means of travel (bus, bike, hovercraft):*Ask us!

**Accommodation/Stay in Prince George**

For the evening prior to our departure (Saturday, [Date]) and/or the evening following our return (Saturday, [Date]), please indicate in the participant information form if you need hotel accommodation in Prince George. We will book this for you. During these times you are welcome to contact other members of the roadshow and arrange an activity/dinner etc. Your meals will be reimbursed at UNBC per diem rates (max. $42 dinner, $18 breakfast). If you want suggestions for things you might do in Prince George do not hesitate to ask the Roadshow team and we will provide as much information as we can.

**Schedule**

A tentative schedule of the week has been provided as an additional attachment in the email. Remember that this schedule is subject to change and you will not receive a finalized copy until the start of the week of your Roadshow.

We will meet at [Time] Sunday, at the medical building (building 9) at UNBC. If you need a ride to UNBC please indicate so on the participant information form.

We aim to return to Prince George in the evening on Saturday, [Date] at approximately [Time].

**Costs**

All scheduled accommodations, meals, and activities are organized and covered by the program. Anything you choose to partake in outside of the scheduled time and/or any alcohol you purchase is not covered. If at any time you are required to purchase meals or other amenities keep your receipts as you may be reimbursed for these at the end of the Roadshow.

**School Presentations**

We are visiting [x number of] schools in [Communities], and will be doing multiple presentations at each.

For each presentation, there will first be a ~12 minute Powerpoint talk to a large audience (approximately 60-80 youth) in a school gymnasium or multi-purpose room. You are asked to create and deliver one slide of the Powerpoint presentation explaining who you are, why you have chosen to study in your chosen healthcare profession, and what you enjoy about it. A powerpoint template will also be attached to this email. Each student will have one minute to present. We highly recommend including pictures!

***Some general thoughts on preparing your one minute presentation:***

- remember that one minute goes by quick, so keep it short and sweet.
- practice a couple times so that you get a good flow down and have the timing right.
- use your life stories if you think they enhance your presentation but if you want to keep it more generalizable then that is good as well.
- a slide with too many words is impossible for the kids to read, especially in one minute, a couple sentences is fine, but try not to put in three or four large paragraphs.
- overall, remember why you are there, to inspire these kids to think your career choice is “cool” and to inspire them to pursue healthcare and academia in general.
- Your presentation doesn’t need to beat out Charlie Chaplin’s speech in The Great Dictator, sometimes simple is better

***An example presentation speech:***

“Hi everyone, my name is [Blank] and I am going into my second year in the Northern Medical Program. I was born and raised in Prince George, BC so being able to go to school at UNBC is really amazing. I originally wanted to go into medicine when a couple family members of mine passed away from cancer. I thought to myself ‘I want to go into oncology, I’m going to help people beat cancer’. After volunteering for a while in a clinic though, I realized that any healthcare career can make a huge impact on a patient’s life, and the life of their loved ones. It’s a privilege to be able to work with patients and their families. The reason that I love medicine is because I really love being responsible for knowing the science of medicine, and explaining it to patients. My classmates are an amazing group of diverse people that really support me when I need it. We aren’t all just nerds hanging out in the library every day. Maybe the most important thing I can tell you though is that I was a terrible high school student. So regardless of who you are or what people tell you, you can do anything you chose to. Thank you.”

Ideally you are striving to be yourself, to be relatable, to say why you chose your career with something **specific** that is **positive**, is more than just wanting to help people (that’s all of healthcare), and is not disparaging towards any other healthcare career (we’re not in competition against one another).

After the Powerpoint, each healthcare career will have their own station. Students will rotate through each station in small groups (6-8 youth), spending 6-8 minutes at each station. At your healthcare station, you will deliver an interactive activity/presentation using healthcare equipment related to your field of study (e.g. anatomy models, crutches, wheelchair, microscope, x-ray light box, birthing model, etc.).

We will provide equipment related to your field to the best of our abilities, but feel free to bring any equipment of your own – just let us know in advance. If you accrue extra travel expenses because of bringing equipment with you please keep the receipts and we will reimburse you for the cost.

***A few notes about the small group presentations:***

- remember that you are trying to keep the kids engaged. Giving them something to do often works better than simply talking to them.
- Each group of students is different, some might not be interested in your station, or in the Roadshow in general. Don’t feel discouraged (they are teenagers after all). However, if you feel like students are not engaging well with your station feel free to ask for feedback from the roadshow team, we might be able to give some suggestions on fun things you can do with the students.
- Familiarize yourself with your equipment, especially if you are borrowing it from the roadshow team.
- If you find one student is particularly interested in your profession and wants to speak to you after the session is finished that is great. Remember though that if you find you do not have enough time to explain everything about the education process or your profession’s scope of practice giving them a couple good websites/resources to visit, a pamphlet or someone to contact can be super helpful.
- six to eight minutes can be really long or really short depending on how you structure your activity. While we may wish to have all the time in the world with these kids we have to keep in mind that our mandate is to give everyone an equal opportunity to see each station. To that effect try to respect time constraints.

**Expectations**

We have found that the Roadshow is most successful when participants are committed to engaging fully in the week’s activities. As such, we expect participants to remain with the team throughout the week. This means participating in ice breakers, activities and dinners. Of course, if you become ill please let us know and we can excuse you from certain activities.

Remember that you are also considered a representative of both your profession, as well as the Healthcare Travelling Roadshow team as a whole, as such please conduct yourself professionally (within reason).

In order to ensure that we do not have students getting lost and to avoid undesirable situations please inform a member of the Roadshow Team of where you are going and who you are going with if you choose to explore the community outside of the scheduled tours.

We strongly encourage all of you to take a look at the Healthcare Travelling Roadshow website at: <https://www2.unbc.ca/healthcare-travelling-roadshow>. This will give you some ideas about the goals of the roadshow, what to expect during this week and how to structure your presentations.

**Appendix 5: Participant Information Form**

Healthcare Travelling Roadshow [Year]

Participant Information Form

| 1. **Participant Information** | | | | |
| --- | --- | --- | --- | --- |
| Full name:      *(if you will be flying, this needs to be* ***exactly*** *as on your travel ID)* | | Program and year of study: | | Institution: |
| Address: | | | Date of birth: | |
| Cell number: | | E-mail address: | | |
| Medical conditions we should be aware of: | | Allergies: | | Dietary restrictions: |
| Emergency contact name and relationship: | | | Emergency contact phone number: | |
| 1. **Travel and Accommodation** | | | | |
| Do you need transportation to Prince George for the start of the Roadshow?      *(if you answered No, skip to section 3)* | | | | |
| Mode of travel to/from Prince George:    ☐ Car  ☐ Air travel  ☐ Other (please specify): | Preferred flights (if applicable):  *Please include departure city, flight number, and date/time*    Arrival:        Return: | | | |
| Do you require hotel accommodation in Prince George the night before the Roadshow (Saturday, Date)?    ☐ Yes  ☐ No | | | Do you require hotel accommodation in Prince George the night following the Roadshow (Saturday, Date)?      ☐ Yes  ☐ No | |
| Do you require a ride to UNBC on the morning of Sunday, Date?    ☐ Yes  ☐ No | | | | |
| 1. **Other** | | | | |
| Are you bringing presentation equipment? If so, please specify. | | | | |
| Is there any other information you feel is necessary for us to know prior to your participation in the roadshow? If so, please specify. | | | | |

**Appendix 6: Sample Planning Timeline**

**Roadshow Planning Timelines**

|  | September | | October | November | December | January | February | March | April | May |
| --- | --- | --- | --- | --- | --- | --- | --- | --- | --- | --- |
| Communities | | Plan communities to visit based on expressed interest and/or last visit. | |  |  |  |  |  |  |  |
| Schools | | Connect with schools, ensure there is interest, determine dates that work based on our plans and the school schedule. | | | Finalize school visit schedule |  | Distribute recruitment poster to schools. | | Confirm arrange-ments with schools. |  |
| Transportation | |  | Initial charter booking & cost estimates. | |  |  | Finalize charter details & costs. |  |  |  |
| Recruitment | |  |  | Build applicant survey. | | Advertise opportunity to post-secondary institutions. | | Adjudicate applicants & offer positions. | |  |
| Accommodation/ Dining | |  |  |  |  | Book accommodation for group. | | Finalize accomm-odation | Book restaura-nts |  |
| Healthcare facility tours | |  |  |  |  |  | Plan hospital/healthcare facility tours in 2 - 3 communities, aiming for different types of facilities. | | |  |
| Recreation | |  |  |  |  |  | Plan recreational opportunities, in consultation with community contacts. If possible, plan for a chance to meet civic leaders. | | |  |
| Equipment | |  |  |  |  |  |  | Communicate with participants and programs about equipment needs. Borrow equipment - return after Roadshows complete. | | |
| Schedule | |  |  | Draft schedule (dates, locations and school visits). | |  |  | Schedule finalized and shared with team. | | Roadshow runs! |

**Appendix 7: Sample equipment list**

**Potential Equipment List by Healthcare Career**

** This is a suggested list only based on past Roadshow experience. Any equipment can be used by any profession as seen fit.

| **Profession** | **Equipment** |
| --- | --- |
| Medicine | - Stethoscopes - Reflex hammers - Neuron model - Body model - Arm model - Lung model - Lung specimens - Other tissue specimens - Intubation model (+ laryngoscopes, endotracheal tubes, bag valve mask) - Pen light |
| Nursing | - BP cuffs - Heart model - Stethoscopes - Pen light - Patient monitor - Wound care kit (gauze, bandages) - Cast care kit - IV model* - Stoma kit |
| Occupational Therapy | - Sock aid - Modified utensils and dishes - Splint/Braces (e.g. dynamic split/brace, dorsal extension hand brace, dynamic finger flexor brace, leather wrist working splint, thumb splint) - Reacher (long handled) - Squishy ball - Fiber optic light - Cane, walker, wheelchair, crutches |
| Physical Therapy | - Dynamometer - Rubber disc - Spine model (with deformity) - Wobble boards - Knee model - Foot model - Immobilization WB Boot - AFO Thermamold Plastic - Knee brace - Shoulder pulley - Green theraband - Transfer belt - Wobble cushion - Bosu ball |
| Massage Therapist | - Spine model - Body model - Skeleton with muscle insertion points - Human skull - Muscle model/poster |
| Dental Hygienist | - Jaw and teeth development model - Tooth model - Tooth x-rays - Light box - Dental tools - Dento-forms |
| Dentist | - Skull model - Dental drill - Explorer - Carving tools (e.g. hollenback, discoid/cleoid) |
| Lab Tech | - Microscopes - Histopathology slides (demo slides) - Parrafin tissue blocks - Slide preparation kits - Chemistry tubes - Urinalysis dipsticks - Microbiology plates - Swabs, inoculation loops - Histology, microbiology books |
| X-Ray Tech | - Light box - Demo X-rays (e.g. pre and post surgery, firearm accidents, traffic accidents) - Bone/joint model - Full skeleton |
| Midwifery | - Birthing model - Pregnant abdomen model - Placenta model - Weigh sling - Breast models (regular, lactating) |
| Audiology | - Ear model - Hearing test equipment - Hearing aids - Decibel counter - Tuning fork |
| Speech Language Pathology | - Voice box model - SLP books, games, videos, etc. - ½ Head and neck model - Brain model - Portable U/S |
| Respiratory Therapy | - Intubation model/intubation equipment - Oropharyngeal airway - Nasopharyngeal airway - Bag valve mask |

**Appendix 8: Sample School/Community information poster**
